# *Bacillus* Strains as Effective Biocontrol Agents Against Phytopathogenic Bacteria and Promoters of Plant Growth

**DOI:** 10.1007/s00248-024-02384-1

**Published:** 2024-05-27

**Authors:** José Abrahán Ramírez-Pool, Berenice Calderón-Pérez, Roberto Ruiz-Medrano, Randy Ortiz-Castro, Beatriz Xoconostle-Cazares

**Affiliations:** 1 Departamento de Biotecnología y Bioingeniería, CINVESTAV, Av. Instituto Politécnico Nacional 2508, Mexico City, 07360 Mexico; 2 Unidad de Biotecnología y Prototipos, Facultad de Estudios Superiores Iztacala, Universidad Nacional Autónoma de México, Tlalnepantla, Mexico Mexico; 3 Red de Estudios Moleculares Avanzados, Instituto de Ecología A.C., Carretera Antigua a Coatepec 351, Xalapa, Veracruz 91073 Mexico

**Keywords:** *Bacillus*, Phytopathogen biocontrol, Quorum sensing inhibition, Plant growth promotion

## Abstract

Modern crop production relies on the application of chemical pesticides and fertilizers causing environmental and economic challenges. In response, less environmentally impactful alternatives have emerged such as the use of beneficial microorganisms. These microorganisms, particularly plant growth-promoting bacteria (PGPB), have demonstrated their ability to enhance plant growth, protect against various stresses, and reduce the need for chemical inputs. Among the PGPB, *Bacillus* species have garnered attention due to their adaptability and commercial potential. Recent reports have highlighted *Bacillus* strains as biocontrol agents against phytopathogenic bacteria while concurrently promoting plant growth. We also examined *Bacillus* plant growth-promoting abilities in *Arabidopsis thaliana* seedlings. In this study, we assessed the potential of various *Bacillus* strains to control diverse phytopathogenic bacteria and inhibit quorum sensing using *Chromobacterium violaceum* as a model system. In conclusion, our results suggest that bacteria of the genus *Bacillus* hold significant potential for biotechnological applications. This includes developments aimed at reducing agrochemical use, promoting sustainable agriculture, and enhancing crop yield and protection.

**Supplementary Information:**

The online version contains supplementary material available at 10.1007/s00248-024-02384-1.

## Introduction

Crop production relies on the application of chemical pesticides, fertilizers, and other inputs to protect plants from pathogens and increase yield [1–4]. Recently, there has been a development of less environmentally impactful alternatives. Beneficial microorganisms have been demonstrated to effectively promote plant growth and development while providing protection against both biotic and abiotic stress [5]. Among these soil bacteria that offer benefits to plants are the genera *Acetobacter*, *Azospirillum*,* Azotobacter*, *Bacillus*, *Burkholderia*, *Klebsiella*, *Pseudomonas*, and *Serratia*, collectively termed as plant growth-promoting bacteria (PGPB) [6, 7]. *Pseudomonas* and *Bacillus* are the most prevalent genera, attributed to their adaptability to various environmental conditions [8]. Notably, biological products based on *Bacillus* strains have exhibited the potential to enhance crop yields by up to 40% [8]. Among the commercially utilized *Bacillus* strains, *B. subtilis* and *B. amyloliquefaciens* stand out [9].

Furthermore, there is evidence to suggest that free-living microorganisms in the rhizosphere modulate plant auxin and cytokinin signaling pathways, subsequently leading to alterations in root architecture and improved nutrient absorption. The *Bacillus* genus encompasses soil isolates that can enhance plant growth by increasing auxin and cytokinin levels and modifying root structure [10]. Additionally, *B. thuringiensis* (Bt) and *B. cereus* produce insecticidal crystalline proteins (Cry) during sporulation. Utilizing these proteins can reduce the reliance on chemical insecticides, lower production costs, and minimize environmental impact, thereby promoting a more sustainable and economically viable agricultural model [10, 11].

On the other hand, selected PGPBs protect plants from a range of pathogens and pests, including bacteria, fungi, viruses, and nematodes [12–14] while the precise mechanisms underlying this protection are not yet fully understood. Bacteria have the ability to communicate each other through production of signal molecules as *N*-acyl-homoserine lactones (AHLs) depending on cell density. This process has been named as quorum sensing (QS) modulating diverse functions as growth, biofilm formation, and virulence factor production [15]. Besides, bacteria have the ability to modulate virulence through the production of lactonases. These enzymes can degrade the QS signals, a process known as quorum quenching (QQ). In this context, the *aiiA* gene, which encodes a lactonase capable of degrading AHLs, has been identified in several *Bacillus* strains, acting as quorum sensing inhibitors (QSI) or quorum quenchers [16]. Indeed, the application of a *Bacillus* spp. lysate to a *Pseudomonas aeruginosa* culture resulted in a remarkable 93% inhibition of biofilm formation [17].

In this study, we evaluated different *Bt* strains as potential biocontrol agents against three phytopathogenic bacteria. We also assessed their QS inhibition activity using the *Chromobacterium violaceum* bacteria, a natural indicator strain that produces violacein, a pigment which production by *C. violaceum* is under control of the QS system. Furthermore, we examined the plant growth-promoting and developmental abilities of *Bacillus* strains. Our results have significant implications for the development of biotechnological applications aimed at reducing the use of persistent agrochemical products in agriculture, thereby enhancing crop yield and protection in a sustainable manner.

## Materials and Methods

### Bacterial Strains and Growth Conditions

Fourteen different *Bt* strains were employed. Ten of them were obtained from the National Collection of Microbial Strains and Cell Cultures (CDBB) of Cinvestav, and four from our laboratory strain collection (Table 1). *Chromobacterium violaceum* was provided by the Environmental Microbiology and Phytopathology Laboratory from the Institute of Ecology (Inecol). *Clavibacter michiganensis*, *Ralstonia solanacearum*, and *Xanthomonas campestris* were kindly provided by the National Phytosanitary Reference Center (CNRF), Mexico. All bacterial strains were activated and routinely grown in Luria Bertani (LB) medium and incubated in a shaker at 120 rpm and 28 °C, otherwise indicated.

**Table 1:** Bacterial strains used in the present work

No.	Scientific name	Serovar/laboratory ID	Source*	Observations
1	*Bacillus thuringiensis*	*Bt* R1	Laboratory strain collection	Isolated from agriculture soil
2	*B. thuringiensis*	*Bt* R2	Laboratory strain collection	Isolated from agriculture soil
3	*B. thuringiensis*	*Bt* R3	Laboratory strain collection	Isolated from agriculture soil
4	*B. thuringiensis*	*Bt* R4	Laborator strain collection	Isolated from agriculture soil
5	*B. thuringiensis*	*kurstaki* strain 1	CDBB	Large parasporal crystals
6	*B. thuringiensis*	*kurstaki* strain 2	CDBB	Large parasporal crystals
7	*B. thuringiensis*	strain 1	CDBB	Fast sporulation
8	*B. thuringiensis*	*kurstaki* strain 3	CDBB	Large insecticidal crystals
9	*B. thuringiensis*	strain 2	CDBB	Fast sporulation
10	*B. thuringiensis*	*alesti*	CDBB	Large parasporal crystals
11	*B. thuringiensis*	*entomocidus*	CDBB	Large insecticidal crystals
12	*B. thuringiensis*	*kenyae*	CDBB	Large parasporal crystals
13	*B. thuringiensis*	*aizawai*	CDBB	Large parasporal crystals
14	*B. thuringiensis*	*tolworthi*	CDBB	Large parasporal crystals
15	*Clavibacter michiganensis*		CNRF	-
16	*Ralstonia solanacearum*		CNRF	-
17	*Xanthomonas campestris*		CNRF	-
18	*Chromobacterium violaceum*		INECOL	-

*CDBB Collection of Microbial Strains and Cell Cultures. *CNRF *National Phytosanitary Reference Center. *INECOL* Institute of Ecology, A.C.

### Detection of the AHL Lactonase (*aiiA*) Gene in *Bt* Strains

To investigate the presence of the AHL lactonase (*aiiA*) gene in Bt strains, we performed the following steps: Genomic DNA from Bt strains was isolated following the method described by [18]. The purity and concentration of the extracted DNA was determined by a NanoDrop One spectrophotometer (Thermo Scientific, Waltham MA, USA), and DNA integrity was verified by agarose gel electrophoresis. Amplification of the *aiiA* gene was performed using the forward and reverse specific primers: *aiiA*F (5′-ATGGGATCCATGACAGTAAAGAAGCTTTAT-3′) and *aiiA*R (5′-GTCGAATTCCTCAACAAGATACTCCTAATG-3′), respectively [19]. PCR was carried out using 5 ng of genomic DNA and Platinum Taq DNA polymerase High Fidelity (Invitrogen, Carlsbad CA, USA) commercial system following the manufacturer’s instructions. The reaction conditions were 94 °C for 1 min, 30 cycles at 94 °C for 10 s, 54 °C for 30 s, and 72 °C for 50 s, with a final extension at 72 °C for 3 min. 16s rRNA gene was used as endogenous control using the forward and reverse specific primers: Bakt_341F (5′-CCTACGGGNGGCWGCAG-3′) and Bakt_805R (5′-GACTACHVGGGTATCTAATCC-3′), respectively [20]. PCR conditions were 94 °C for 1 min, 30 cycles at 94 °C for 10s, 52 °C for 30s, and 72 °C 50s, with a final extension at 72 °C for 3 min. The amplified products were then confirmed through agarose gel electrophoresis.

### Phylogenetic analysis of *aiiA* gene

The amplified products of the *aiiA* gene from 11 *Bt* strains were cloned into the pDrive vector (Qiagen, Germantown, MD). Resulting plasmids were purified using the alkaline lysis method [18] and sequenced at LABSERGEN capillary sequencing service unit of LANGEBIO, Cinvestav, Mexico. Sequences were edited with the SeqTrace software v. 9.0 [21]. Basic Local Alignment Search Tool (BLASTn) from NCBI (https://blast.ncbi.nlm.nih.gov/, accessed 15 June 2023) was used to compare gene sequences for AHL lactonase. The nucleotide sequences of *aiiA* gene from *B. thuringiensis* are available in the GenBank database under the following accession numbers according to the serovar or strain: *kenyae* (OR271315), *aizawai* (OR271316), *tolworthi* (OR271317), *kurstaki* strain 1 (OR271318), *kurstaki* strain 2 (OR271319), *kurstaki* strain 3 (OR271320), *alesti* (OR271321), *entomocidus* (OR271322), Bt-R2 (OR271323), Bt-R1 (OR271324), and Bt-R4 (OR271325). The *Bt* sequences of the *aiiA* gene were translated using EMBOSS Transeq (https://www.ebi.ac.uk/Tools/st/emboss_transeq/, accessed 05 July 2023) with bacterial codon usage. The sequences used for the construction of the phylogenetic tree were those obtained in this work and those used by Noor et al. [22]. The obtained sequences were aligned with MUSCLE using MEGA X. The phylogenetic tree was constructed based on the amino acid sequences with the neighbor-joining method using 1000 bootstrap replicates.

### Confrontations of *Bt* Strains Against Phytopathogenic Bacteria

*Bt* strains which amplified for the lactonase gene were grown in liquid LB medium 24 h before the confrontation assay. Each pathogenic bacterium was grown in LB medium, *X. campestris* (24 h), *C. michiganensis* (48 h), and *R. solanacearum* (72 h) and then mixed with soft agar (1%) at a final bacterial concentration of 30% (approximately 2.4 × 10^5^ cells/mL). This soft agar mixture was laid over a solid LB base. Subsequently, different volumes (5, 10, and 20 µL) of *Bt* cultures of approximately 2.4 × 10^5^ cells/mL were added. After incubation at 28 °C for 48 h, the growth inhibition halos were measured. Three independent replicates were performed for each assay.

### Quorum Sensing Inhibition Assays

Quorum sensing (QS) inhibition was screened and evaluated by confronting *Bt* strains or *Bt* extracts with *Chromobacterium violaceum* bacteria. For QS inhibition screening, LB medium containing *C. violaceum* at a final concentration of OD600 = 0.1 (optical density at 600 nm) was poured onto Petri dishes and allowed to solidify. Subsequently, 1.2 × 10^4^ cells/mL of each Bt strain culture were spotted over *C. violaceum* and LB plates (as growth control). After an incubation at 28 °C for 48 h, inhibition halos were observed. For QS inhibition evaluation, five *Bt* strains which produced inhibition halos over *C. violaceum* plates were grown in 150 mL of LB for 48 h. Cultures were centrifuged at 9000 rpm at 4 °C for 10 min. To obtain *Bt* extracts, cell culture supernatants were extracted with 1 volume of sodium acetate. The organic extracts were rotary evaporated at 45 °C and 250 mbar in a R-100 rotavapor (Büchi, Laguna Hills, CA) and resuspended in methanol at a final concentration of 26.8 mg/mL. For the QS inhibition assay, solid LB containing OD_600_ = 0.1 of *C. violaceum* was prepared and 15 µL of *Bt* extracts was spotted. After incubation at 28 °C for 72 h, QS was evaluated through the measurement of inhibition halos. Three replicates were used for each assay.

### Plant Growth Promotion Assay

To evaluate potential effects of *Bt* strains on in vitro co-cultivated plants, *Arabidopsis thaliana* (Col-0) and transgenic lines *ARR5::uidA* [23] and *DR5::uidA* [24] transgenic lines were used. These recombinant lines are used as cytokinin and auxin indicators, respectively. *Arabidopsis* seeds were surface sterilized as described [25] and grown in Murashige and Skoog (MS) medium in a growth chamber at 26 °C, under a 16 h/8 h light/dark photoperiod and 60% relative humidity. *Bt* strains were grown in solid LB (24 h) and inoculated by streaking on plates containing MS medium. Immediately, 6-day-old *Arabidopsis *seedlings were placed on the bacterial streak. Co-cultures were grown for a further 10-day period in the growth chamber. Three replicates were used for each assay. Different growth parameters such as primary root length (cm) and lateral root number and histochemical GUS activity were determined and compared to control seedlings without bacterial strains.

### Histochemical Determination of Reporter Beta-Glucuronidase (GUS) Activity

The reporter gene *uidA* gene of *Escherichia coli*, encoding the enzyme β-glucuronidase (GUS), was employed in the present study. To detect its activity, transgenic seedlings were immersed in a solution of 5-bromo-4-chloro-3-indolyl-β-D-glucuronide (X-Gluc) in 100 mM (pH 7) sodium phosphate supplemented with 2 mM H_4_Fe(CN)_6_ and 2 mM K_3_Fe(CN)_6_ at 37 °C and incubated overnight. Tissue clearing was performed by incubating the seedlings in 2 mL solution of 20% (v/v) methanol and 0.24 N HCl at 60 °C for 45 min. After the removal of the solution, 7% NaOH was added to cover the seedlings and maintained at room temperature for 30 min. The solution was discarded and the seedlings were washed with 40%, 20%, and 10% (v/v) ethanol for 30 min each, and finally stored with 50% glycerol (v/v) at 4 °C [26]. Plant tissues were fixed on glass slides with 50% (v/v) glycerol and representative images were taken using Nomarski optics in a Leica DM5000B microscope at 20× magnifications.

### Statistical Analysis

Ten plants for each treatment were employed, assayed in three replicates. To identify differences between multiple groups’ means while controlling the experiment-wise error rate, the data obtained were statistically analyzed by average comparison and one-way ANOVA (analysis of variance) with Tukey’s post hoc test for multiple comparisons using SAS software and graphed with the GraphPad Prism v. 8.0.

## Results

### Detection and Phylogenetic Analysis of the AHL Lactonase (*aiiA*) Gene in *Bt* Strains

**Fig. 1:**
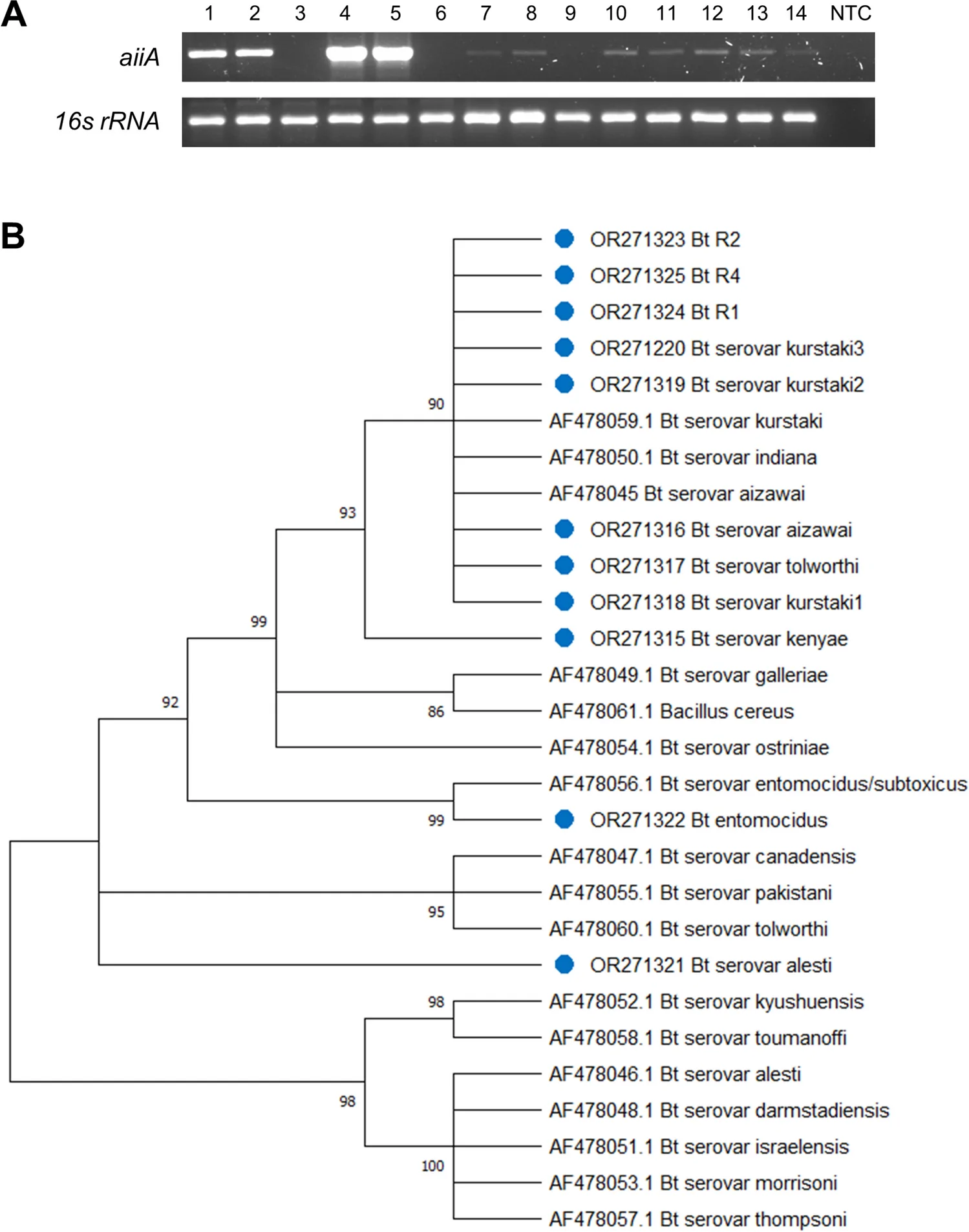
Detection and phylogenetic analysis of the lactonase gene in *Bt* strains. **A** PCR detection of *aiiA* (lactonase, 750 bp) and *16s rRNA* (endogenous, 450 bp) genes from genomic DNA of different *Bt* strains. 1: *Bt* R1; 2: *Bt* R4; 3: *Bt* R3; 4: *Bt* serovar *kenyae*; 5: *Bt* serovar *aizawai*; 6: *Bt* strain 1; 7: *Bt* serovar *tolworthi*; 8: *Bt* serovar *kurstaki* strain 1; 9: *Bt* strain 2; 10: *Bt* serovar *kurstaki* strain 2; 11: *Bt* serovar *kurstaki* strain 3; 12: *Bt* R2; 13: *Bt* serovar *alesti*; 14: *Bt* serovar *entomocidus*; NTC, non template control. **B** Phylogenetic analysis based on the amino acid sequences of the lactonase with the neighbor-joining method using 1000 bootstrap replicates

While it is known that Bt produces various antimicrobial molecules such as bacteriocins, lipopeptides, and cell wall hydrolytic enzymes [27], our study aimed to investigate if Bt strains could disrupt the quorum sensing (QS) of phytopathogenic bacteria. Therefore, we first tried to detect in the *Bt* strains the lactonase encoding gene (*aiiA*), an enzyme that catalyzes the lactone-ring opening of AHL inactivating the QS signal and production of virulence factors. A 750-bp PCR product was amplified from 11 out of the 14 *Bt* strains analyzed (Fig. 1A). The *aiiA* gene could not be amplified in Bt R3, Bt strain 1, and Bt strain 2 using the specified primers, warranting further investigation into the reasons for this lack of amplification. However, amplified *aiiA* gene from the other 11 *Bt* strains were cloned and sequenced to confirm their identity. The obtained sequences were deposited in the GenBank database under the accession numbers OR271315 to OR271325. We conducted a phylogenetic analysis using genes encoding *Bacillus* lactonases (Fig. 1B) to understand the relationships and similarities among different strains. Eight *Bacillus* strains (OR271323, OR271325, OR271324, OR271320, OR271319, OR271316, OR271317, and OR271318) were clustered with Bt serovar kurstaki, indiana, and aizawai lactonases, demonstrating high similarity, while *Bt* serovar *kenyae* (ORF271315) was more distant but derived from the same node. On the other hand, the lactonase sequence OR271322 was clustered with *Bt* serovar *entomocidus* and *Bt* serovar *alesti* (OR271321) was situated on an external branch.

### *Bt* Strains Inhibited Phytopathogenic Bacterial Growth

**Fig. 2:**
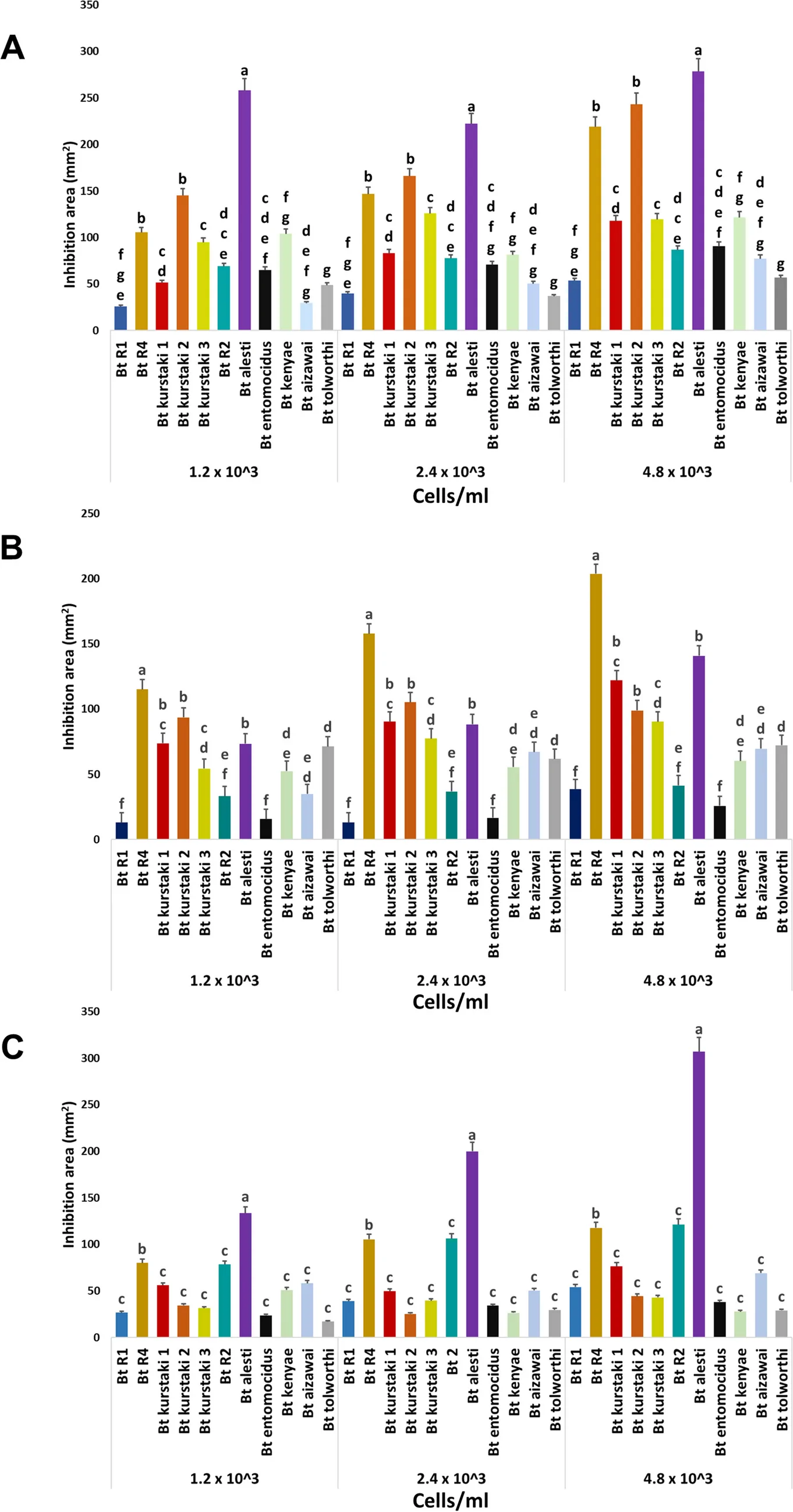
Confrontation assays of *Bt* strains against phytopathogenic bacteria. Inhibition area (mm^2^) of growth inhibition of phytopathogenic bacteria treated with different concentrations of *Bt* strains (1.2 × 10^3^, 2.4 × 10^3^, and 4.8 × 10^3^ cells/mL) in Petri dishes. Confrontation against **A ***C. michiganensis*, **B ***R. solanacearum*, and **C ***X. campestris.* Standard deviation bars are shown (three replicates were evaluated). Different letters indicate statistically significant differences between groups, as determined by the Tukey test (*p* < 0.05) for each cell concentration

The 11 *Bt* strains in which *aiiA* gene was amplified were tested for their capacity to inhibit the growth of phytopathogenic bacteria (including *X. campestris*, *R. solanacearum*, and *C. michiganensis*). Growth inhibition halos were measured in Petri dishes from the confrontation assays. All *Bt* strains showed apparent inhibition activity against phytopathogenic bacteria, some of them in a concentration-dependent manner (Fig. 2). *Bt* serovar *alesti* showed the highest inhibition against *C. michiganensis*, followed by *Bt* serovar *kurstaki* strain 2 and *Bt* R4 (Fig. 2A). In the case of *R. solanacearum*, *Bt* R4 exhibited the best results inhibiting this phytopathogenic bacteria followed by *Bt* serovar *alesti* and *Bt* serovar *kurstaki* strain 2 (Fig. 2B). Finally, in the confrontation assay with *X. campestris*, the highest inhibition of growth was observed with *Bt* serovar *alesti* followed by *Bt* R4 and *Bt* R2 (Fig. 2C).

### *Bt* Strains Disrupted Quorum Sensing

**Fig. 3:**
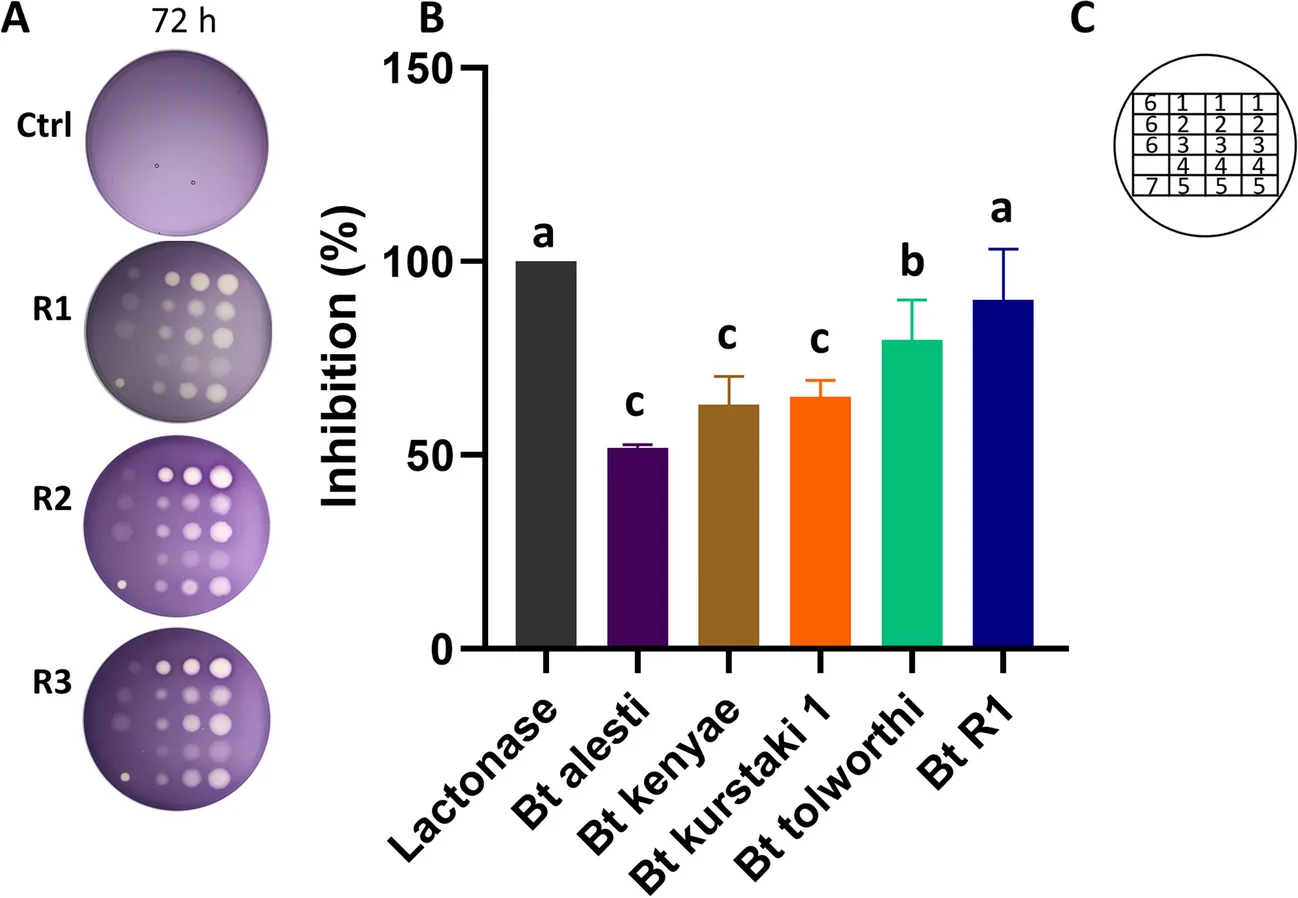
Quorum sensing inhibition evaluation in *C. violaceum* treated with *Bt* extracts. **A** Plates showing inhibition of violacein synthesis at 72 h post-inoculation (hpi). Ctrl, control; R1-3, replicates. **B** Quantification of violacein synthesis inhibition after 72 hpi treatment with 0.4 mg of *Bt* extracts, contained in 15 µL. Different letters indicate statistically significant differences determined by the Tukey test (*p* < 0.05). **C** Inset indicating the location of *Bacillus* assayed by triplicate: 1. *Bt* R1; 2. *Bt kenyae*; 3. *Bt tolworthi*; 4. *Bt alesti*; 5. *Bt kurstaki* strain 1; 6. methanol (negative control); 7. acyl-homoserine lactone (positive control)

*Bt* strains in which *aiiA* gene was amplified were also tested for their ability to disrupt QS. For this purpose, *C. violaceum* was used as the accumulation of the compound violacein occurs when bacterial population reaches a high density [28]. For QS inhibition screening, 11 *Bt* strains were tested and five of them were selected based on the presence of inhibition halos (Table S1). Then, the extracts of these *Bt* strains were assayed for their capacity to inhibit violacein synthesis. The halos representing the absence of violacein were quantified to determine if the extracts, containing lactonase, disrupted *C. violaceum* QS as described in [28]. All the *Bt* strain extracts inhibited violacein synthesis, although *Bt* R1 produced the largest inhibition halos compared to the purified lactonase (positive control) (Fig. 3). Statistical analysis of QS inhibition evaluated by triplicate formed a group with the positive control and *Bt* R1, followed by *Bt tolworthi*, which showed 74% inhibition of violacein synthesis, compared to the negative control with methanol. *Bt kurstaki* strain 1, *Bt kenyae*, and *Bt alesti* isolates grouped together showing from 49 to 60% QS inhibition.

### *Bt* Strains Promoted Plant Growth

**Fig. 4:**
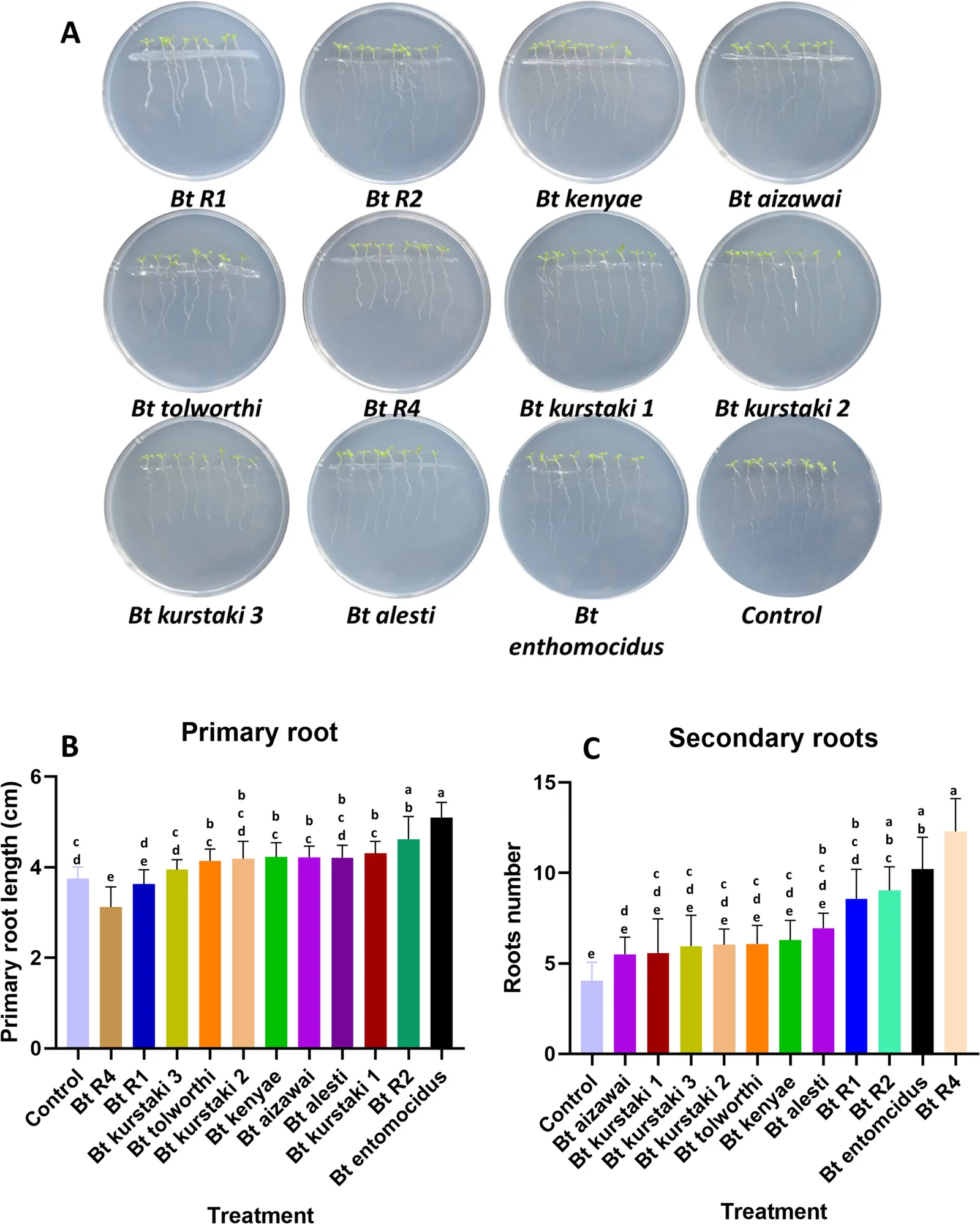
Effect of *Bt* strains on root system architecture in ARR5 *Arabidopsis* seedlings. **A** Petri dishes with the plant-bacteria interaction. *Arabidopsis* seedlings were inoculated with different *Bt* strains. Control indicates non-inoculated plates with *Bt* strains. **B** Primary root length (cm). **C** Lateral root number. Different letters indicate statistically significant differences determined by the Tukey test (*p* < 0.05)

To evaluate the ability of *Bt* strains in the plant growth promotion, *Arabidopsis* seedlings were used as an experimental model. Six-day old *Arabidopsis *seedlings were co-inoculated on a bacterial streak and growth further ten days. Primary root length and lateral secondary root number were determined for each treatment (Fig. 4). The root architecture in all treatments consisted of a main root and lateral roots with different number and length. Likewise, the development of normal photosynthetic cotyledonary leaves was observed (Fig. 4A). Statistical analysis showed that the treatments were clustered in five groups based on the effect on primary root growth and architecture. *Bt entomocidus* and *Bt* R2 induced primary root growth with statistical significance compared with the control. On the other hand, there was a statistically significant decrease in the primary root length with *Bt* R4. The other *Bt* strains did not show statistical differences compared with the control treatment (Fig. 4B). For secondary root number, *Bt* R4 produced the highest amount followed by *Bt entomocidus*, *Bt* R2, and *Bt* R1. The other *Bt* strains performed similar to the control treatment (Fig. 4C). In a parallel assay, *Arabidopsis* seedlings were co-inoculated with pathogenic bacteria (Fig. S1). Interestingly, *X. campestris* and *C. michiganensis* increased primary root length and induced formation of secondary roots with statistical significance, comparable to some *Bt* strains. In contrast, *R. solanacearum* failed to induce primary root growth, but significantly increased secondary root number.

### *Bt* Strains Induced the Expression of Auxin and Cytokinin Responses in *Arabidopsis* Seedlings

**Fig. 5:**
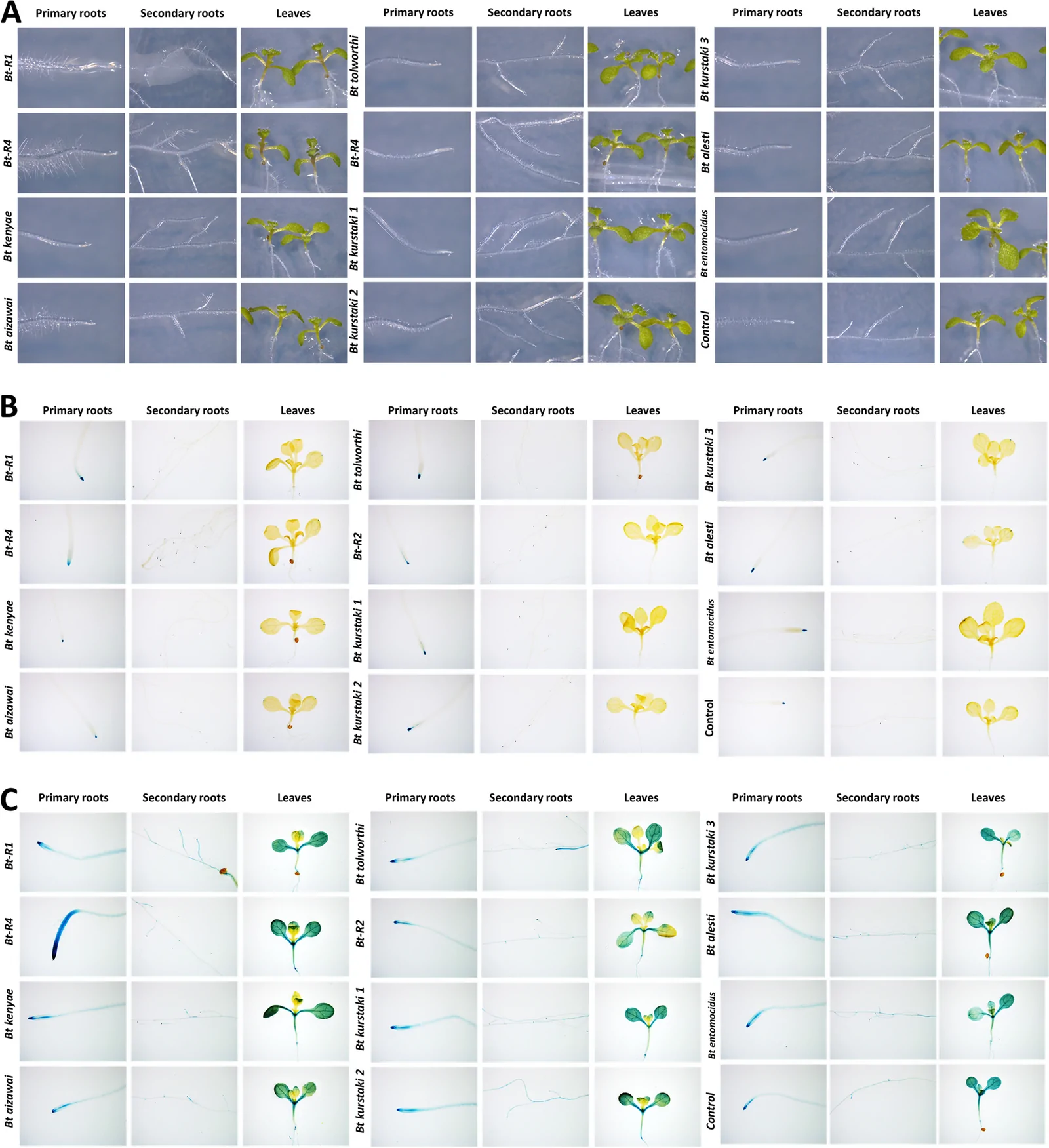
Effect on auxin and cytokinin accumulation in *Arabidopsis* seedlings inoculated with *Bt* strains. **A** Representative images illustrating the impact of different *Bacillus* strains on the growth of primary roots, secondary roots, and shoots in *Arabidopsis* seedlings. Afterwards, seedlings were stained for GUS activity. **B** *Arabidopsis* expressing the auxin inducible construct *DR5::uidA* treated with the *Bt* strains. **C** *Arabidopsis* expressing the cytokinin inducible promoter *ARR5::uidA* treated with the indicated *Bt* strains. Representative images of analyzed plants are shown

As shown, plant-bacteria interaction resulted in modifications to root architecture, which may be mediated by auxins and cytokinins. To explore this possibility, we assayed *Arabidopsis* line reporter seedlings harboring the synthetic DR5 auxin responsive element directing the expression of the GUS reporter gene [24], and a second *Arabidopsis* expressing the GUS reporter gen under the control of the ARR5 pseudo-response regulator gene promoter responsive to cytokinins [29]. Increased accumulation of auxins and cytokinins was monitored by histochemical GUS staining of seedlings treated with different *Bt* strains (Fig. 5). *Bt* R1, *Bt* R2, and *Bt alesti* treatments led to the highest number of root hairs, which directly contribute to nutrient absorption (Fig. 5A). Plant-bacterial interactions with *DR5::uidA* showed higher levels of auxin accumulation after treatment with *Bt* R1, *Bt* R2, *Bt tolworthi*, *Bt alesti*, *Bt kurstaki* strain 1, and *Bt kurstaki* strain 2 (Fig. 5B). The accumulation of auxins in leaf tissue was observed in the interactions with *Bt* R1, *Bt* R2, *Bt kenyae*, and *Bt entomocidus.* In contrast, auxin accumulation was undetectable in leaves of control plants, and only observed in the apical region of the primary root. On the other hand, transgenic line *ARR5::uidA* seedlings with *Bt* R2, *Bt kurstaki* strain 1, and *Bt entomocidus* induced cytokinin accumulation in apical roots, stems, and leaves (Fig. 5C). Of note is the differential cytokinin accumulation in leaves of the treated plants, which showed a patchy distribution, in contrast to control plant tissue which showed a uniform accumulation pattern of the reporter gene. Parallel experiments were conducted with both plant transgenic lines and pathogenic bacteria (Fig. S2). The strongest auxin induction was observed with *R. solanacearum*, in which the *Arabidopsis* seedling developed thickened roots, as well as a greater number of hairy roots displaying strong GUS expression. The accumulation of auxins in leaf tissue was observed in the interaction with *C. michiganensis* and *R. solanacearum* (Fig. S2).

## Discussion

The transition towards sustainable agriculture has amplified the interest in exploring alternatives to chemical fertilizers and pesticides. Plant bio-stimulating microorganisms encompass bacteria that either facilitate plant growth and development or protect crops against pests and diseases [30]. Among these, numerous bacterial species within the *Bacillus* genus are recognized as PGPB. The mechanisms governing PGPB activity involve the production of plant growth regulators, enhanced nutrient uptake from the soil, and the secretion of deterrent factors against phytopathogenic bacteria [31]. Notably, *Bacillus* species produce a variety of antimicrobial agents, such as bacteriocins and defensins, which exhibit effectiveness against a broad spectrum of gram-negative bacteria [32].

Conversely, QS is a mechanism that enables bacteria to adapt to environmental stress, including extreme temperatures and nutrient scarcity. QS is also associated with bacterial virulence, flagella synthesis, aerobic respiration, resistance to toxins and oxidative stress, biofilm formation, and the production of toxins, effector molecules, extracellular enzymes, and polysaccharides [22, 33]. Furthermore, the ability to disrupt QS, known as QQ, is a widespread strategy employed by bacteria across various domains of life, allowing them to compete with other bacteria and deter bacterial infections in eukaryotes. Inhibition of QS in phytopathogenic bacteria may offer a highly effective alternative for disease control. *Bacillus*, in particular *B. thuringiensis*, has been reported to produce insecticidal proteins [34], as well as antibacterial compounds and lactonases that are instrumental in restricting growth of gram-negative bacteria by hydrolyzing the diffusible signals that govern QS [35].

To assess the potential QS inhibition activity in *B*. *thuringiensis *strains, we initially attempted to amplify the lactonase gene from bacterial genomic DNA. The identification of *Bt* strains harboring sequences (ORF271315 to ORF271325) bearing a high similarity to *aiiA* genes encoding lactonases suggested their potential to disrupt QS. Phylogenetic analysis indicates a high degree of homology among lactonases in several *Bacillus* strains, implying a conserved role. This notion is reinforced by studies revealing that a mutated lactonase (H109Y) exhibits reduced activity compared to wild-type lactonases, highlighting a degree of specialization in *Bacillus* QS disruption [22, 35].

Subsequently, 11 *Bt* strains in which the *aiiA* gene was amplified were tested for their ability to inhibit phytopathogenic bacteria, specifically *R. solanacearum, X. campestris*, and *C. michiganensis*. The selection of these strains was based on their demonstrated pathogenicity and virulence. *R. solanacearum* infects over 200 plant species worldwide, including potato (*Solanum tuberosum*), tomato (*Solanum lycopersicum*), and banana (*Musa *spp.) [17, 36]. *Xanthomonas *spp. infect approximately 400 plant species, including sugar cane, beans, cassava, cabbage, banana, citrus, tomato, chili pepper, and rice [37]. Their life cycle involves epiphytic and endophytic stages, with the latter marked by biofilm formation in the plant’s vascular system, enhancing their pathogenicity [38, 39]. *C. michiganensis* is a gram-positive bacterium that infects the xylem of plants and causes significant agricultural losses [40]. Notably, *C. michiganensis* regulates its QS via cyclic peptides (oligopeptides) [41, 42], suggesting that *Bacillus*-mediated QQ for this bacterium occurs through secreted proteases targeting these peptide signals.

*C. violaceum*, which serves as a model for direct QS-mediated violacein synthesis inhibition [21, 28], was used to screen and evaluate the inhibition of QS by *Bt* strains. The statistical analysis revealed that the selected strains effectively inhibited QS. *Bt* R1 and *Bt tolworthi* displayed 90% and 74% inhibition, respectively, while *Bt* kurstaki strain 1, *Bt* kenyae, and *Bt* alesti exhibited inhibition percentages ranging from 49 to 60%. These results suggest that *Bt* strains have the potential to mitigate damage to agricultural crops caused by these pathogenic bacteria. The inactivation of genes associated with QS (*rpfF*, *rpfC*, and *RpfG*) significantly reduced the virulence of *Xanthomonas citri*, which infects grapefruit leaves [43]. Conversely, overexpressing the *Xylella fastidiosa rpfF* gene in Carrizo citrange plants intensified the damage caused by *X. citri* at inoculation sites, indicating that heterologous QS signals disrupt signaling [44]. These findings underscore the role of QS in pathogenicity and virulence during infection and lay the foundation for using QS inhibition and QQ to control phytopathogenic bacteria.

Regarding the assessment of *Bt* strains as PGPB, *Bt* *entomocidus* and *Bt* R2 were found to promote growth and development in *Arabidopsis* seedlings, particularly in terms of primary root length. *Bt* R4, on the other hand, resulted in the highest number of secondary roots, followed by *Bt* *entomocidus*, *Bt* R2, and *Bt* R1. Additionally, *Bt* R1, *Bt* R2, and *Bt alesti* treatments led to the highest number of root hairs, which directly contribute to nutrient absorption. These findings align with previous reports on the advantages of the use *Bacillus* as PGPB and microbial plant biostimulants (MPBs) [45]. For example, various *Bacillus*-based formulations have been applied to crops such as tomato, canola, wheat, saffron, soybean, potato, apple, cassava, and tobacco, resulting in increased production and protection against pathogen attacks [46]. However, our results also indicate that *X. campestris* and *C. michiganensis* promote root growth and development, potentially due to auxin and cytokinin production by these bacteria. Speculatively, this could be a strategy employed by the bacteria to expand the infection area of their hosts. However, in healthy plants, the induction of auxin and cytokinin responses is clearly beneficial considering the promotion of growth, including the root production to increase water and mineral absorption.

*Arabidopsis* root architecture changed in response to *Bacillus* treatment, evinced by the induction of lateral (secondary) roots. Plants produced lateral roots as a part of their natural growth and development, playing a crucial role in supporting the plant’s overall structure, anchoring it to the substrate, and enhancing nutrient and water absorption. The production of lateral roots is regulated by auxins, such as indole-3-acetic acid (IAA), responsible for promoting their initiation, cell elongation, and differentiation. The differences in lateral root induction by *Bacillus* isolates may reflect the efficiency in the crosstalk with plant cells, yet to be investigated. Multiple PGPB produce auxins and cytokinins, enabling them to form a close association with plant roots by increasing the root surface and absorption area [47]. In this study, we tested this phenomenon using reporter genes for auxin and cytokinin response. The results revealed a notable induction of the auxin reporter gene, indicating auxin accumulation following interaction with *Bt* R1, *Bt* R2, *Bt tolworthi*, *Bt alesti*, *Bt kurstaki* strain 1, and *Bt kurstaki* strain 2. Moreover, *Bt* R2, *Bt kurstaki* strain 2, and *Bt entomocidus* demonstrated a higher accumulation of cytokinins. A suitable crosstalk between auxin and cytokinin pathways is crucial for stem elongation, root development, and response to pathogen infections. In cases involving the interaction of *R. solanacearum*, high auxin accumulation was observed in leaf tissue, but plants displayed stunted development. This could potentially be a strategy employed by these bacteria to expand their infection area [48]. Furthermore, the three phytopathogenic bacteria studied exhibited elevated levels of cytokinin accumulation, which may not be strictly necessary for the host but could facilitate the infection process. It has been reported that other *Bacillus* species produce phytohormones in vitro, including abscisic acid (ABA) and gibberellins (GA4 and GA3), which confer tolerance to both biotic and abiotic stress [49].

The successful use of beneficial microorganisms hinges on their adaptability to various agroecosystems, encompassing differences in soil, climate, and crop types. Thus, the application of native soil microorganisms to safeguard crops against phytopathogenic bacteria in agriculture holds promise. According to our findings, *Bt* strains can serve multiple purposes in intensive agriculture, from defending against pathogen attacks to promoting plant growth. This multifaceted approach has the potential to reduce the environmental impact of excessive agrochemical application.

*B. thuringiensis* exerts significant influences on plant growth through yet to be discovered induction of phytohormone synthesis such as auxins, gibberellins, and cytokinins, controlling plant growth, including cell elongation, root development, and overall biomass production. Additional research is needed to understand how *Bacillus* strains induce the expression of genes related to plant hormone signaling pathways to further influence the plant’s physiological responses. This intricate interplay between *Bacillus* and phytohormones highlights the potential of these bacteria as biostimulants for promoting sustainable and robust plant growth in agricultural and horticultural settings.

Crop protection involving PGPB can complement modern biotechnological strategies within the framework of sustainable agriculture. This may include the use of genetically edited plants with reduced water consumption or increased drought tolerance via CRISPR/Cas9 gene editing, without compromising yield [50], or the engineering of plants to resist pathogen attacks, as seen in the case of Huanglongbing affecting citrus worldwide [51]. Other options include the use of nanoparticles loaded with antimicrobials for controlling various phytopathogens [52]. These alternatives should be considered part of integrated crop management.

The use of PCPB in sustainable agriculture is desirable because it is environmentally friendly; however, in a risk assessment of its use, the concern on the potential development of resistance in pathogens over time is possible. To consider a long-lasting use of such microorganisms, we should consider the selective pressure, the natural evolution of the pathogens to overcome competition with biocontrollers, the use of diverse combination of such PCPB to reduce resistance, the incorporation of different strategies to integrated pest-management (cultural practices, resistant crop varieties, and chemical control), and a regular monitoring of plant growth and pathogen population for timing adjustment. In summary, while the potential for resistance development exists, careful management practices, including the use of diverse biocontrol agents and integrated approaches, can help mitigate this risk and maintain the efficacy of biocontrol in sustainable agriculture.

We employed *B. thuringiensis* strains to enhance plant growth and provide protection against bacterial pathogens. Our study demonstrated that the inhibition of quorum sensing by these *Bacillus* strains is associated with increased plant vigor. The present research proposes the use of beneficial microorganisms as an environmentally friendly alternative. While the reduction of agrochemical use is desirable for promoting sustainable agriculture and enhancing crop yield, it presents new challenges. Potential future directions for research in this field could be focused in assessing its use under agricultural environments, optimizing the large-scale production of *Bacillus*-based products with stringent quality control, and their effective integration into plant management by producers worldwide.

## Supplementary Information

Below is the link to the electronic supplementary material.

Supplementary file1 (DOCX 10.9 MB)

## Data Availability

The nucleotide sequence data reported are available in the GenBank database under the accession numbers OR271315 to OR271325.

## Code Availability

The following software were used: Seqtrace software v. 9.0, BLASTn (https://blast.ncbi.nlm.nih.gov/, accessed 15 June 2023), EMBOSS Transeq (https://www.ebi.ac.uk/Tools/st/emboss_transeq/, accessed 05 July 2023), MUSCLE using MEGA X v. 10.1.8, SAS software (https://welcome.oda.sas.com/ accessed 31 July 2023), and GraphPad Prism v. 8.0.
