# Supplementary material for: Bacillus Strains as Effective Biocontrol Agents Against Phytopathogenic Bacteria and Promoters of Plant Growth
Source: Microb Ecol. 2024 May 27;87:76. doi: 10.1007/s00248-024-02384-1 (PMC11129970; doi:10.1007/s00248-024-02384-1)
Supplement: Supplementary file 1 [file 248_2024_2384_MOESM1_ESM.docx]

**Supplementary Material**

**Supplementary tables**

**Table S1** Screening of quorum sensing inhibition of *C. violaceum* with *Bt* strains.

| ***B. thuringiensis* (*Bt*) strain** | **Inhibition halo** |
| --- | --- |
| *Bt* R1 | Present |
| *Bt* R2 | Absent |
| *Bt* R4 | Absent |
| *Bt kurstaki* strain 1 | Present |
| *Bt kurstaki* strain 2 | Absent |
| *Bt kurstaki* strain 3 | Absent |
| *Bt alesti* | Present |
| *Bt entomocidus* | Absent |
| *Bt kenyae* | Present |
| *Bt aizawai* | Absent |
| *Bt tolworthi* | Present |

**Supplementary figures**

**Fig. S1** Effect of plant pathogenic bacteria on primary root length and secondary root number in ARR5 Arabidopsis seedlings. **A** Petri dishes with the plant-bacteria interaction. Arabidopsis seedlings were inoculated with *R. solanacearum*, *C. michiganensis*, or *X. campestris*. Control indicates non-inoculated plates. **B** Primary root length (cm). **C** Secondary root number. Different letters mean statistically significant differences with Tukey test (p<0.05).

**Fig. S2** Effect of phytopathogenic bacteria treatment with Arabidopsis seedlings on auxin and cytokinin accumulation. **A** Representative pictures of the effect on Arabidopsis seedlings in the growth of primary roots, secondary roots and shoots, inoculated with different phytopathogenic bacteria. Afterwards seedlings were stained for GUS activity. **B** Arabidopsis expressing the auxin inducible construct *DR5::uidA* treated with the indicated phytopathogenic bacteria. **C** Arabidopsis expressing the cytokinin inducible promoter *ARR5::uidA* treated with the indicated phytopathogenic bacteria. Representative images of analyzed plants are shown. R.s.: *R. solanacearum*, C.m.: *C. michiganensis*, X.c.: *X. campestris.*
